# Extendable intramedullary nailing in a child with osteogenesis imperfecta of bilateral femoral fractures: a case report

**DOI:** 10.3389/fsurg.2025.1454192

**Published:** 2026-01-13

**Authors:** Weishuai Zhang, Xuchao Lu, Nannan Yang, Xianyou Zhu, Haotian Hu

**Affiliations:** 1 The Fifth Ward of Orthopedics Department, Kaifeng People's Hospital, Kaifeng, Henan, China; 2 Department of Anesthesiology, Kaifeng People's Hospital, Kaifeng, Henan, China

**Keywords:** osteogenesis imperfecta, femoral fractures, extendable intramedullary nailing, orthopedic, children

## Abstract

**Background:**

Osteogenesis imperfecta, commonly referred to as brittle bone disease, is the most prevalent monogenic bone disorder and is characterized by osteoporosis and heightened bone fragility. Most patients experience multiple fractures, some of which can be managed conservatively; however, patients with numerous fractures frequently develop significant limb deformities and growth abnormalities that require surgical intervention. For adult patients, intramedullary fixation of osteotomies is typically the preferred option; however, this approach is less suitable for children due to their ongoing growth and development, which necessitates periodic replacement of intramedullary nails and thus repeated surgeries. Moreover, increased bone fragility and a high propensity for fractures in children with osteogenesis imperfecta contribute to frequent postoperative complications, such as refractures and displacement of internal fixation. To address these challenges, extendable intramedullary nails have been developed. In this context, we used these nails to treat a pediatric patient with multiple femoral fractures and severe deformity, with favorable clinical outcomes during a 2-year postoperative follow-up.

**Case report:**

We present the case of a 12-year-old female patient with postnatally diagnosed osteogenesis imperfecta who sustained multiple fractures over time, the most severe being bilateral femoral fractures. She was admitted to our facility on two occasions for femoral shaft fractures. We employed osteotomy, orthopedic techniques, and extendable intramedullary nailing for her treatment. Postoperatively, she showed satisfactory recovery from bilateral femoral deformities, with successful fracture healing and near-normal lower limb lengths.

**Conclusion:**

Extendable intramedullary nailing exhibits favorable clinical efficacy in the management of fracture-related deformities in pediatric patients with osteogenesis imperfecta, offering novel insights and options for clinical diagnosis and treatment, thereby demonstrating significant clinical utility.

## Introduction

Osteogenesis imperfecta (OI), commonly known as brittle bone disease, was first described by W. Vrolik in 1849 as a syndrome characterized by bone fragility and multiple fractures occurring during the prenatal period or immediately after birth (1). OI is a rare autosomal dominant disorder, with an estimated incidence of 1 in 15,000 to 1 in 20,000 individuals (2). Mutations in *COL1A1* and *COL1A2* genes, which encode type I collagen, represent the primary pathogenic mechanism underlying the disease (3). The main clinical features include decreased bone mineral density, increased bone brittleness, developmental abnormalities, blue sclerae, dental abnormalities, hearing loss, impaired lung function, and cardiac valve disease. Some severe forms of OI can be fatal during the perinatal period. In 1979, Sillence et al. classified it into four types based on clinical severity. In 2014, Van and Sillence improved the previous classification by incorporating emerging clinical evidence, proposing an improved Silence classification system (4): type I (mild): increased bone fragility with blue sclera and hearing loss, autosomal dominant; type II (lethal): perinatal lethality, autosomal dominant or autosomal recessive; type III (progressive): perinatal fractures, severe bone loss with progressive scoliosis, progressive hearing loss that may lead to complete deafness, occasional blue sclerae, autosomal dominant or autosomal recessive; type IV (moderate to severe): recurrent fractures with skeletal deformities, typically normal sclerae and hearing, autosomal dominant or autosomal recessive; and type V (severe): recurrent fractures with skeletal deformities, associated with calcification of the interosseous membrane and hypertrophic bone crust formation (due to IFITM5 mutation), autosomal dominant. With the continuous identification of genetic variants, modern nomenclature has been developed, among which the International Nomenclature of Physical Diseases (INCDS) proposed by Skelton is particularly significant. The INCDS classification divides OI into five types. Types 1–4 correspond to the Sillence classification, while type 5 is a newly added OI type characterized by calcification of the interosseous membrane (5). The imaging features and phenotype of type V differ from those of other OI types (Table 1). Currently, the treatment of osteogenesis imperfecta relies mainly on medication and surgical intervention; however, there is no uniform standard for the choice of surgical plan, and treatment remains particularly challenging in pediatric patients with multiple fractures and severe limb deformities.

**Table 1:** Comparison table of osteogenesis imperfecta (OI) classification systems.

Classification system	Type	Clinical features	Genetic basis
Sillence (1979)	I	Mild, increased bone fragility, blue sclerae, hearing loss, autosomal dominant	COL1A1/2 mutations
	II	Lethal, perinatal lethality, autosomal dominant or dominant recessive	COL1A1/2 mutations
	III	Progressive, perinatal fractures, severe bone loss, progressive scoliosis, hearing loss, may have blue sclerae, autosomal dominant or dominant recessive	COL1A1/2 mutations
	IV	Severe, recurrent fractures, skeletal deformities, normal sclerae and hearing, autosomal dominant or dominant recessive	COL1A1/2 mutations
INCDS (2009)	I–IV	Same as Sillence classification types I–IV	Same as Sillence classification types I–IV
	V	Severe, recurrent fractures, skeletal deformities, calcification of the interosseous membrane	IFITM5 mutation, autosomal dominant
Skeleton (Sam and Dharmalingam, 2017)	1 (Traditional I)	Mild, non-deforming: bone fragility, prepubertal fractures, blue sclerae/hearing loss	COL1A1 (rarely COL1A2)
	2 (Traditional II)	Perinatal lethal, intrauterine fractures, postnatal death, dentinogenesis imperfecta	COL1A1/COL1A2
	3 (Traditional III, VI, VIII–IX, X; Bruck I)	Progressive severe, birth fractures, severe scoliosis/shortening, fading blue sclerae, deafness	COL1A1/COL1A2 (III), SERPINF1 (VI), CRTAP/LEPRE1/PPIB (VII–IX)
	4 (Traditional IV, VII, XI–XIII)	Moderate deforming, recurrent fractures, dentinogenesis imperfecta, short stature	COL1A1/COL1A2 (IV), SERPINH1 (X), FKBP10 (XI), SP7 (XIII)
	5 (Traditional V; OPPS; IJO; Bruck I–II)	Moderate, recurrent fractures, forearm calcification, hypertrophic calluses, joint hyperextensibility	IFITM5 (V), LRP5 (OPPS), PLOD2 (Bruck)

## Case description

**Figure 1:**
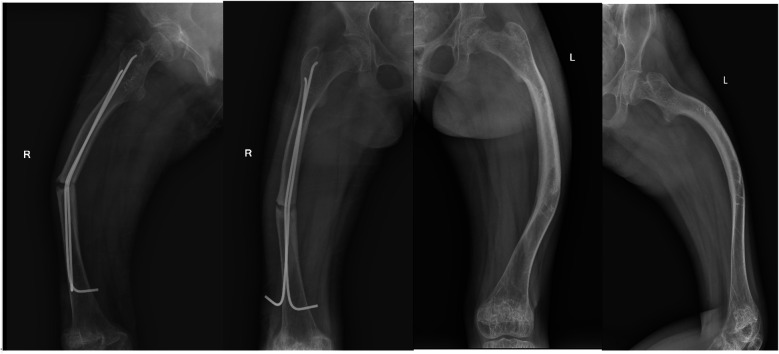
X-ray showing right femoral deformity, left femoral deformity, and remnants of elastic intramedullary nails in the marrow cavity of the right femur.

**Figure 2:**
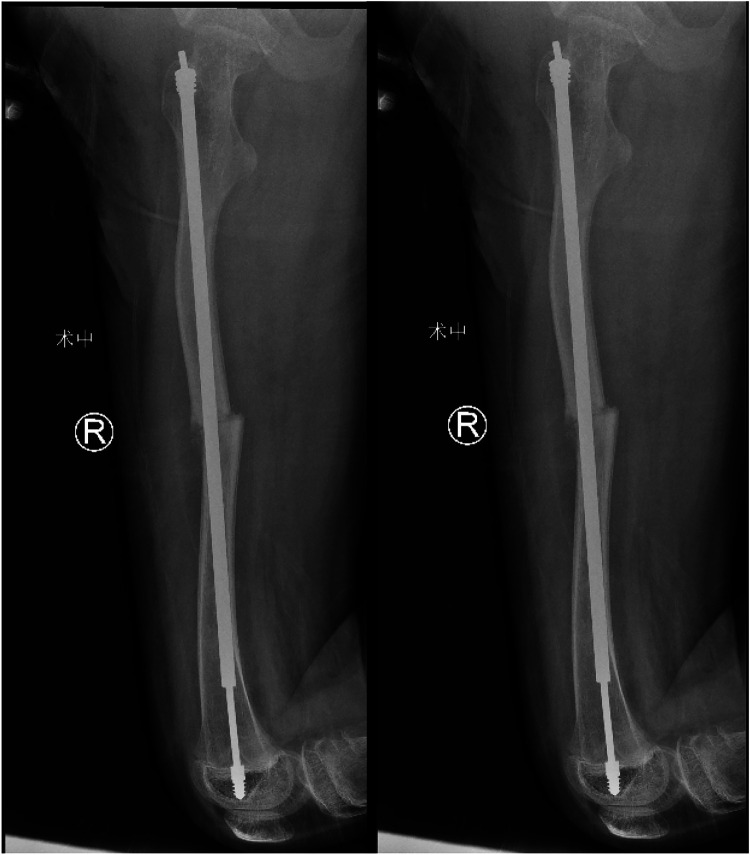
Removal of elastic intramedullary nails with concomitant extendable intramedullary nail internal fixation.

**Figure 3:**
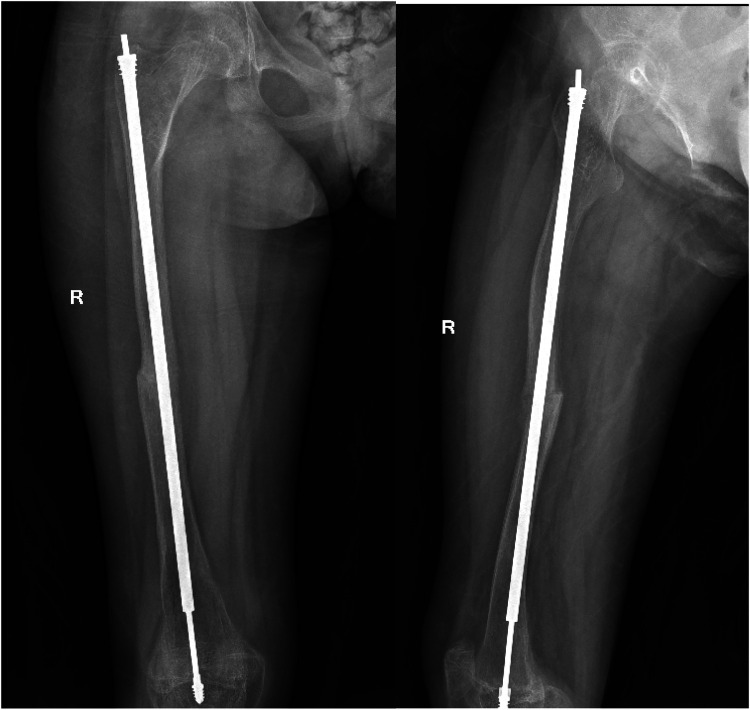
Repeat X-ray at 6 months postoperatively showing a well-healed fracture.

**Figure 4:**
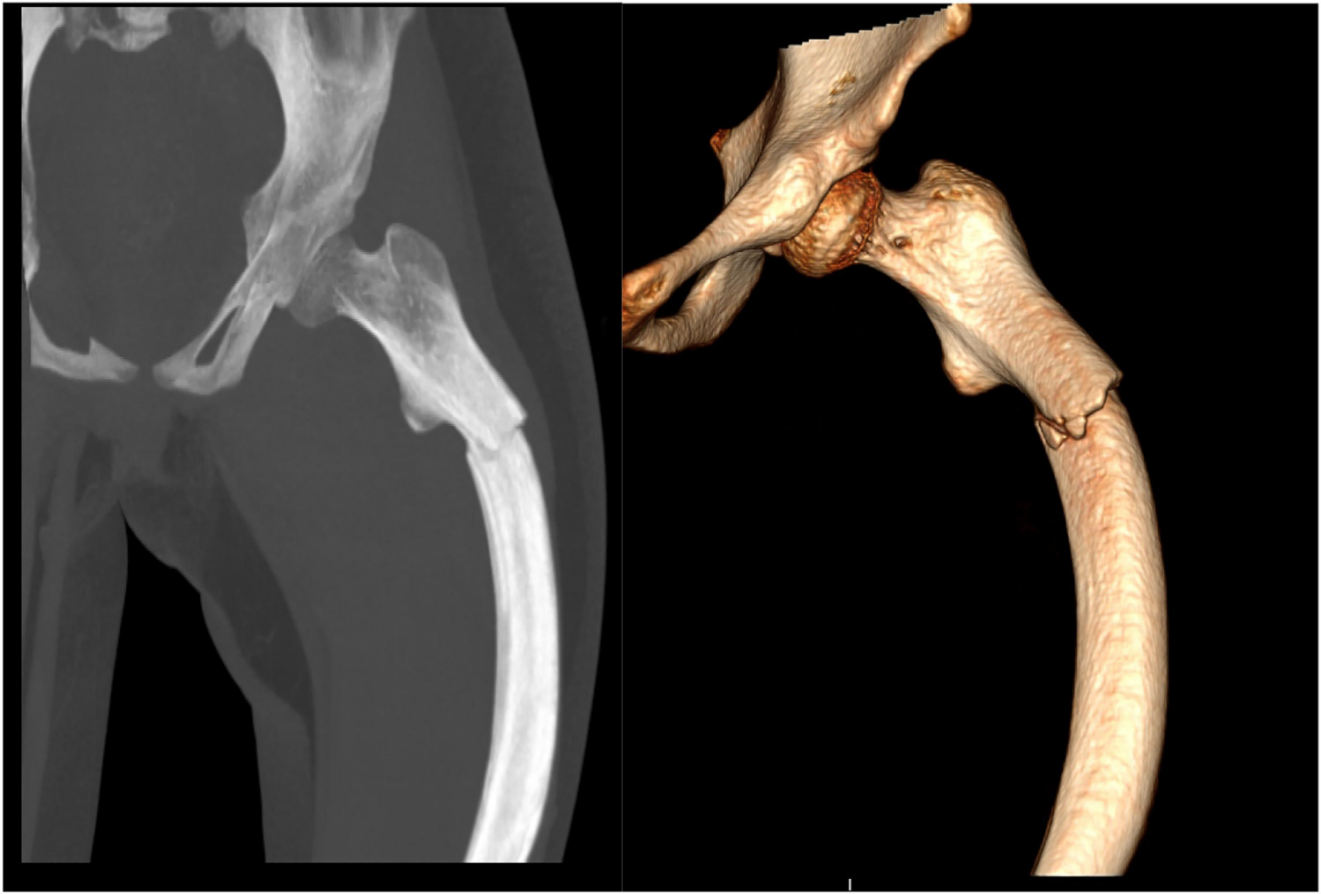
Comprehensive CT 3D reconstruction showing a left proximal femoral fracture, accompanied by severe left femoral deformity.

**Figure 5:**
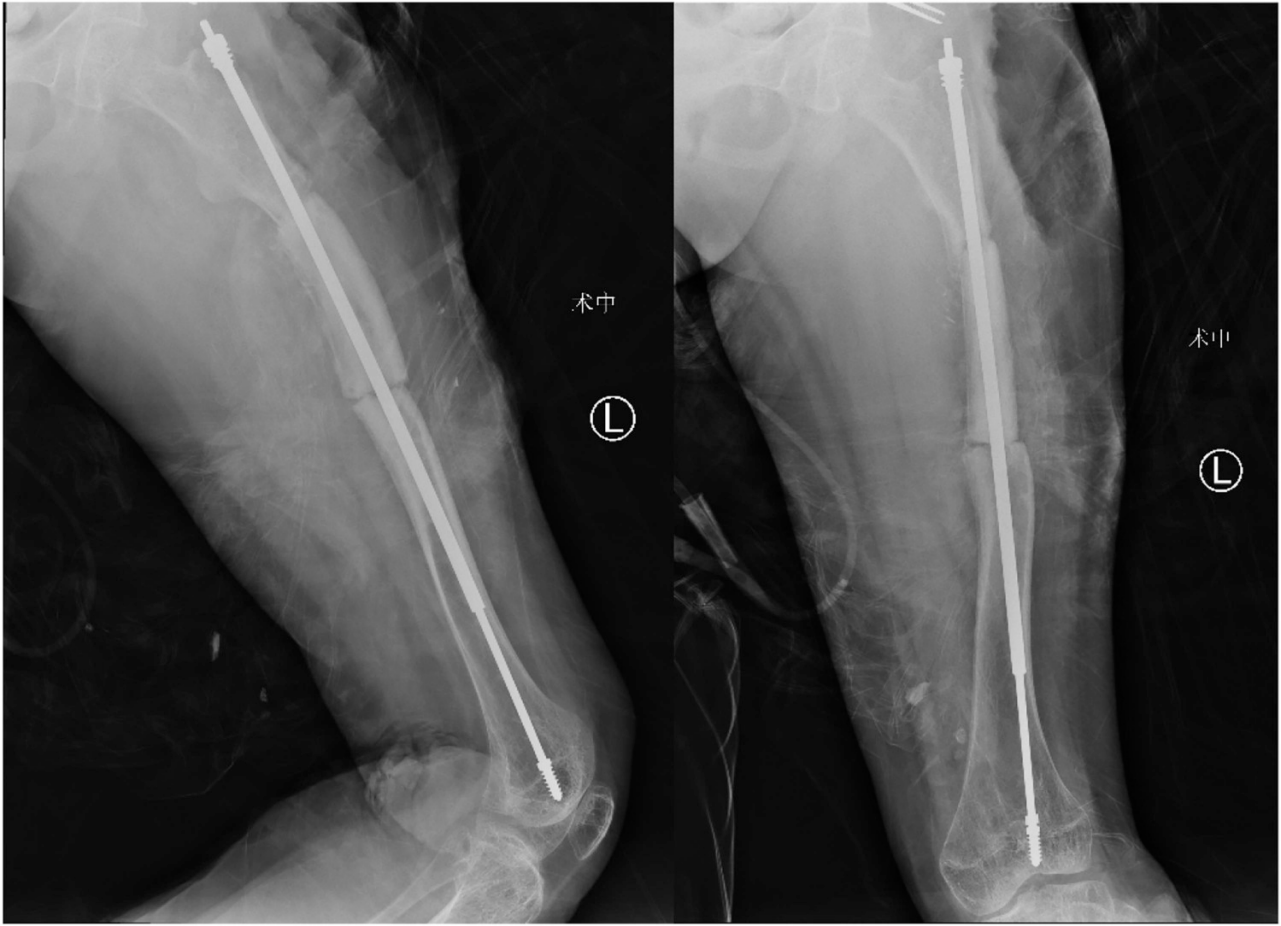
Postoperative review of the X-rays indicating that the left femur deformity had been corrected significantly, and the left lower limb force line recovered well.

**Figure 6:**
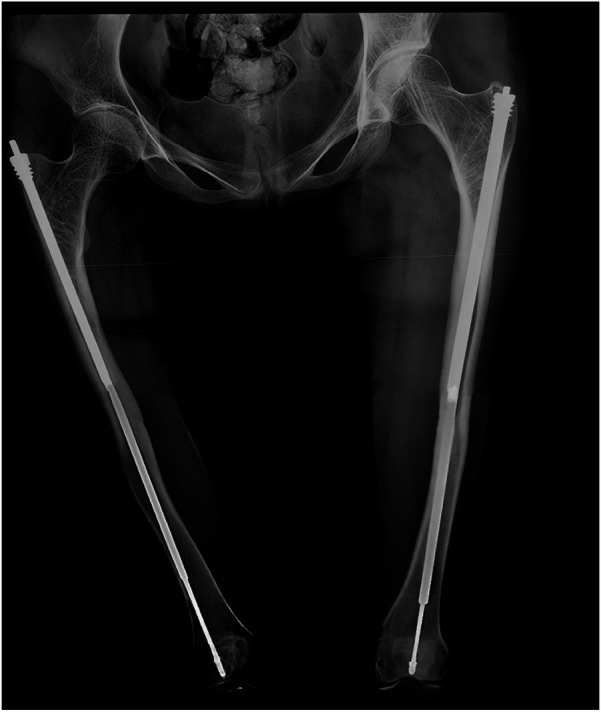
Two years after the operation, it was found that the original bilateral femoral fracture and left femoral osteotomy were completely healed, with restoration of strength in both lower limbs.

**Figure 7:**
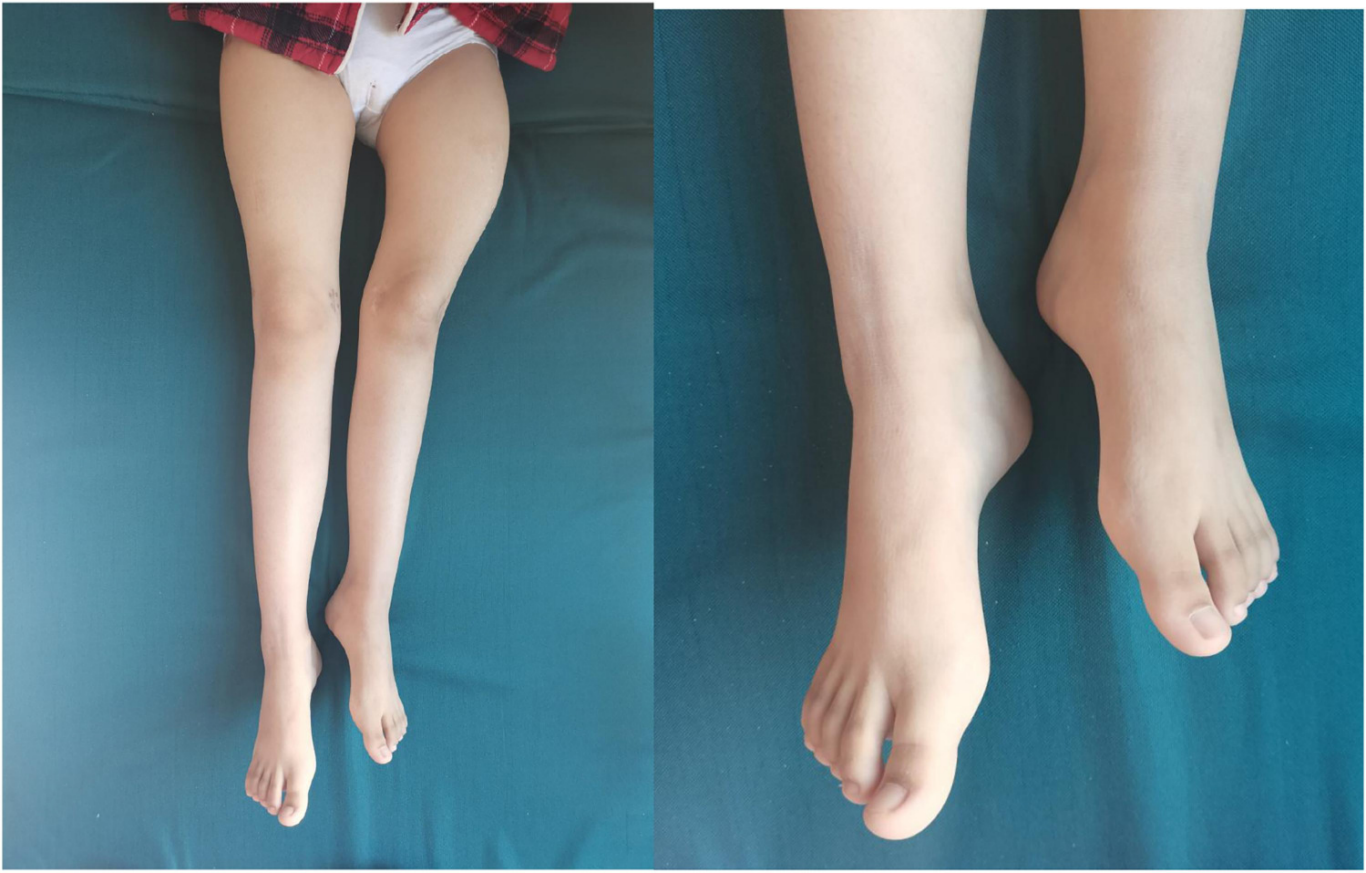
Preoperative shortening of the left lower limb by about 4 cm compared to the right lower limb.

**Figure 8:**
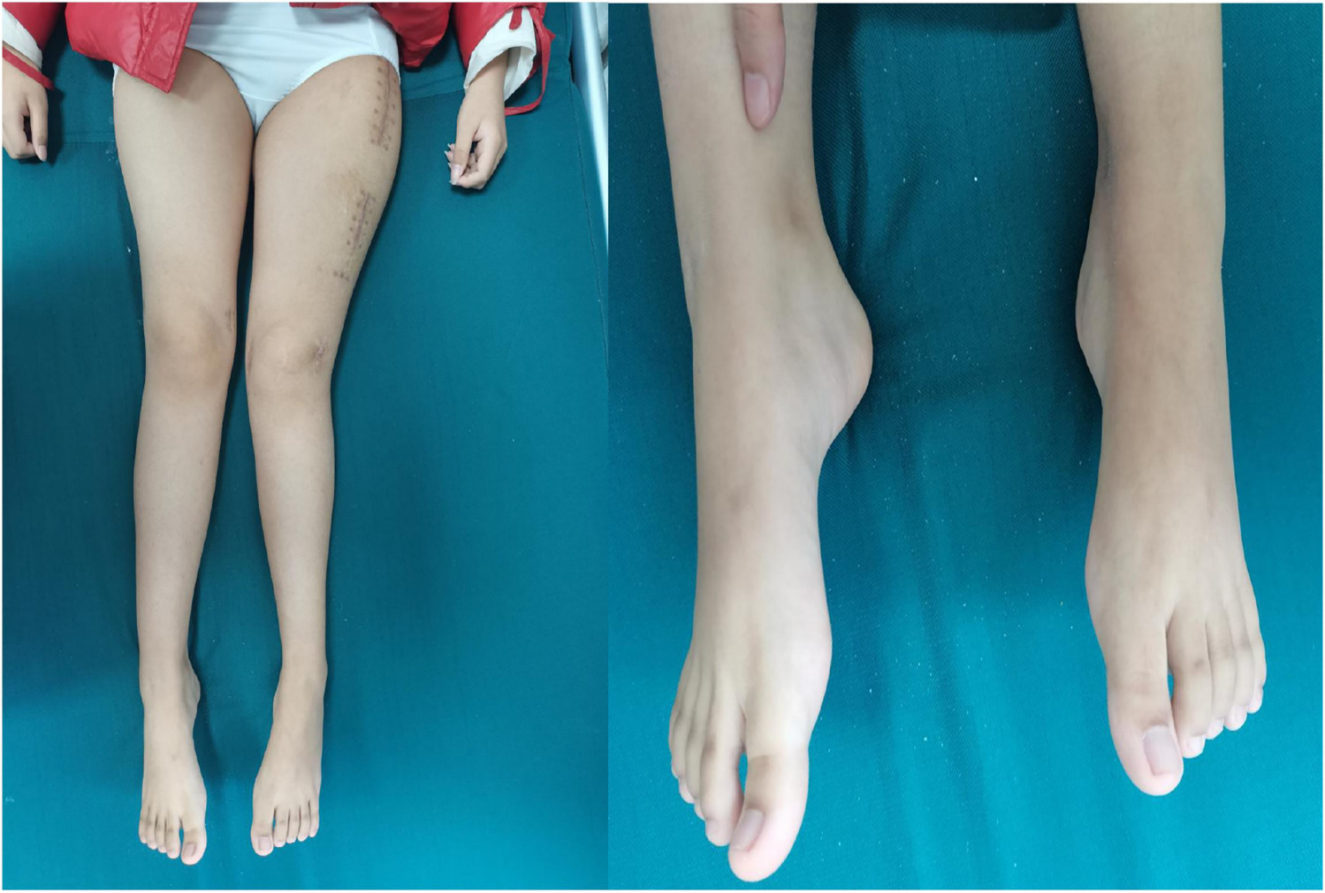
Completely corrected preoperative bilateral lower-extremity inequality.

A 12-year-old girl presented to our hospital after slipping on a slippery surface at home, resulting in pain in the right thigh and restricted mobility. The attending orthopedic surgeon noted that the right thigh was swollen and deformed, along with the presence of blue sclerae. The patient had been diagnosed with osteogenesis imperfecta at birth and had endured numerous fractures throughout her life, including those of both femurs and the tibia, with the right femur being the most frequently fractured. The most recent fracture occurred 2 years prior and was treated at a local hospital with internal fixation using resetting elastic intramedullary pins, resulting in satisfactory postoperative recovery. The patient had not received regular medical treatment for OI, such as bisphosphonate therapy, prior to this admission. Upon admission, radiographic (X-ray) examination revealed a right femoral shaft fracture with residual elastic intramedullary nails in the medullary cavity and marked deformity of the left femur (Figure 1). Relevant examinations showed obvious contraindications to surgical intervention. On the second day after injury, removal of the elastic intramedullary nail, fracture reduction, and internal fixation using an extendable intramedullary nail were performed (Figure 2). The surgery was performed via a lateral femoral approach and lasted 100 min. The total hospital stay was 3 weeks. After 3 weeks of postoperative bed rest, the patient gradually initiated lower-extremity functional exercises. At the 6-month postoperative follow-up, anteroposterior and lateral X-rays of the right femur showed satisfactory fracture healing (Figure 3). One year postoperatively, the patient sustained another fall while walking, resulting in left thigh pain and limited mobility, and was subsequently readmitted to our hospital. Upon admission, comprehensive CT 3D reconstruction revealed a proximal fracture of the left femur accompanied by severe femoral deformity (Figure 4). On the third day after the injury, the patient underwent femur osteotomy and internal fixation with an extendable intramedullary nail. This surgery was also performed via a lateral femoral approach and lasted 150 min. Intraoperative imaging using a C-arm X-ray machine was performed during both surgical procedures to ensure adequate fracture reduction and accurate positioning of the internal fixation devices. Postoperative anteroposterior and lateral X-rays of the left femur showed significant correction of femoral deformity and satisfactory restoration of lower-extremity alignment (Figure 5). At the 2-year follow-up, both the original femoral fractures and the left femoral osteotomy had completely healed (Figure 6). The patient showed good functional recovery of both lower limbs and had fully resumed normal daily activities. Preoperatively, the left lower limb was approximately 4 cm shorter than the right (Figure 7); this limb-length discrepancy was completely corrected following surgery (Figure 8). Given the satisfactory restoration of limb length, appearance, and alignment of both lower extremities, no additional full-length X-rays of the bilateral lower extremities were performed. During the 2-year follow-up, the patient experienced multiple traumatic episodes but sustained no further fractures.

## Discussion

OI exhibits marked genetic heterogeneity, with more than 90% of cases linked to pathogenic variants in *COL1A1* and *COL1A2*, which encode the α1 and α2 chains of type I collagen. Extensive population-based studies by Zhytnik et al. (6) have underscored substantial ethnic variability in the frequency of COL1A1/2 mutations, reported in 63.8% of Ukrainian (7), 86.7% of Estonian (8), and 59.4% of Vietnamese (9) patients with OI. In the present case, genetic testing was not performed at the family's decision; however, based on the patient's clinical features, including blue sclerae, recurrent femoral fractures, and severe skeletal deformities, and in accordance with the Sillence classification criteria, the patient was diagnosed with Sillence type III OI.

To our knowledge, there is no targeted etiologic treatment for osteogenesis imperfecta (10). The clinical goal of medical management is to improve bone structure and strength to reduce fracture risk (11). Medication therapy is primarily aimed at alleviating pain, increasing mobility, and enhancing the quality of life. In patients requiring surgical intervention, preoperative active medical treatment can, to a certain extent, reduce the risk of surgery and improve surgical outcomes. Bisphosphonates, teriparatide, parathyroid hormone, and growth hormone are the preferred medications (12). Among these, bisphosphonates are considered first-line therapy, as they inhibit osteoclast activity, thereby increasing bone mineral density and reducing fracture risk (13). Other agents, such as teriparatide (a parathyroid hormone analog), may stimulate osteoblast activity; however, their use in pediatric patients requires caution due to the potential risk of osteosarcoma in growing bones. Moreover, optimal therapeutic dosages, administration routes, and treatment durations have yet to reach clinical consensus. In addition to the aforementioned drugs, denosumab is currently under investigation for the treatment of OI. It inhibits bone resorption by blocking the RANKL pathway (12), and preliminary studies suggest that it can reduce fracture risk. In addition, gene therapy and stem cell transplantation have been developed in recent years. For example, gene-editing techniques are being explored to correct mutations in the *COL1A1/2* genes, and mesenchymal stem cell transplantation has shown preliminary benefits in improving bone mineral density in animal models and early clinical trials (14, 15). Patients with osteogenesis imperfecta often sustain multiple fractures; however, not all fractures require surgical intervention, particularly in pediatric patients (16). Conservative treatment may be used for fractures without significant displacement, with intervention options (including casts, splints, braces, or traction) tailored to the individual. Although there is little difference between fracture healing outcomes of conservative and surgical treatments, conservative treatment often requires prolonged limb immobilization, which can exacerbate osteoporosis, lead to muscle atrophy and loss of muscle strength, and ultimately increase the likelihood of refracture.

At present, there is no unified standard for the surgical treatment of osteogenesis imperfecta (17, 18). Decisions regarding surgical indications and the choice of surgical strategy are predominantly based on clinical experience. Intramedullary fixation is preferred for long-bone fractures in patients with osteogenesis imperfecta, and elastic intramedullary nailing is commonly used in pediatric patients because of its minimal invasiveness (19), with its effectiveness supported by clinical practice. However, elastic intramedullary nails lack sufficient strength for postoperative fixation and frequently require additional stabilization with traction or plaster, thereby limiting early functional rehabilitation. Karami et al. reported favorable clinical outcomes using the sliding double elastic intramedullary nail technique in children with osteogenesis imperfecta (20). Nevertheless, elastic intramedullary nails do not prevent refracture (21). Interlocking intramedullary nails, with significantly greater fixation strength than elastic nails, are used in heavier patients and can reduce the risk of refracture to some extent (22). However, their application in OI patients is limited by the narrow medullary cavity, and they do not completely eliminate the risk of refracture.

Bone plates represent another option for internal fixation in patients with osteogenesis imperfecta-related fractures and are characterized by eccentric fixation (23). Because patients with osteogenesis imperfecta exhibit marked bone thinning and increased cortical brittleness, the enhanced locking mechanisms and angular stability of locking plates—while improving stability and pullout resistance in osteoporotic bone—do not benefit patients with osteogenesis imperfecta. Stress shielding between the plate and the bone surface can accelerate local bone loss. The incidence of complications, such as screw loosening or breakage at the end of the plate, has been reported to be approximately 15% (24), Although the improved locking mechanisms and angular stability of the locking plate can enhance stability and anti-pullout force to some degree in osteoporotic bone, the risk of internal fixation failure due to screw loosening and pullout remains substantial in patients with osteogenesis imperfecta. Moreover, direct contact between the bone plate and the bone surface results in stress shielding, further accelerating local bone loss and exacerbating osteoporosis (25, 26). Consequently, some authors advocate the use of plates combined with bone allografting by when intramedullary nailing is not feasible due to bone marrow cavity closure or when comminuted fractures require additional stability beyond that provided by intramedullary nail fixation alone. Bone allografting, which involves transplantation of donor bone tissue to areas of bone defects or areas requiring reinforcement, can effectively enhance local bone strength and promote fracture healing (27). However, plate fixation typically requires the placement of multiple screws, and in some cases, additional procedures such as cerclage wiring or bone grafting may be necessary (28). In 1959, Harold Sofield and Edward Miliar first proposed the use of extendable intramedullary nails for the treatment of osteogenesis imperfecta. Subsequently, in 1963, Robert Bailey modified and developed the Dobow–Bailey extendable intramedullary nail, marking a significant progress in the treatment of limb fractures in children with osteogenesis imperfecta (1). This technique helps avoid stress concentration between the nail and bone, accommodates bone growth in pediatric patients, and reduces the risk of refracture (29, 30). However, this method was associated with a high probability of complications, such as displacement of the T-member at the nail ends. Sheffield et al. further improved this extendable intramedullary nail by incorporating T-member fixation to ensure better anchorage within the epiphysis. However, this design requires joint incision during surgery, thereby increasing patient trauma (31). To protect the joint, minimize surgical trauma, and reduce implant displacement, Fassier and Duval subsequently developed a new extendable intramedullary nail (32), featuring an additional nail tail at the distal end, thereby reducing the refracture rate. Their nail also features multiple layers of threads at the distal end, which secure the nail within the epiphysis and prevent rotational displacement. The surgery is conducted solely through a proximal entry incision, thereby minimizing surgical trauma and preserving neighboring joints, while effectively preventing refracture. Yong et al., in a meta-analysis encompassing 594 patients over a 40-year period, proved that extendable intramedullary nails can effectively reduce the incidence of refracture and related complications (33), a finding consistent with the conclusion of Nguyen et al. (34). Hung et al. identified adequate screw insertion depth, central positioning of the nail, and adequate correction during osteotomy as key factors for the successful use of the FD nail (35). Emet et al., considering the complications and revision requirements of children with OI, suggested that intramedullary fixation combined with the plate and screw technique is a more reliable option for the treatment of OI (36).

## Conclusions

Fractures and skeletal deformities are relatively common in children with osteogenesis imperfecta, yet there is no clear consensus regarding optimal treatment strategies or surgical protocols. This case, along with long-term follow-up, demonstrates that FD extendable intramedullary nailing is an effective treatment option in such patients, offering advantages such as minimal surgical trauma, technical simplicity, and reliable clinical outcomes; these findings warrant further clinical research.

## Data Availability

The original contributions presented in the study are included in the article/Supplementary Material, further inquiries can be directed to the corresponding author.
